# Supplementary material for: Goss’s Wilt Resistance in Corn Is Mediated via Salicylic Acid and Programmed Cell Death but Not Jasmonic Acid Pathways
Source: Plants (Basel). 2023 Mar 28;12(7):1475. doi: 10.3390/plants12071475 (PMC10097360; doi:10.3390/plants12071475)
Supplement: Supplementary file 1 [file plants-12-01475-s001.zip › plants-2213983-supplementary.pdf]

Supplementary Table S1. Real time RT-PCR primer sequences.

Gene ID	Primer	Sequence
ZM2G153541	*EF1a* (S)	TGGGCCTACTGGTCTTACTACTGA
	EF1a (A)	ACATACCCACGCTTCAGATCCT
ZM2G067225	*ZmAOS* (S)	CCCAGCGTCGACAACAA
	ZmAOS (A)	TGTCGTAGCGGAGGAAGA
ZM2G101769	*ZmJaz12* (S)	CTGATGCTAAGAAGCCTACTCGC
	ZmJaz12 (A)	GCGTCTGAAGGAGAAGTTTGGTA
ZM2G017616	*ZmLOX9* (S)	CATGGCATCAGACTCCTCATC
	ZmLOX9 (A)	GTAGAGCTGCACATACGACTC
ZM2G118345	*ZmPAL* (S)	TCTGGTCCCGCTCTCCTACA
	ZmPAL (A)	TTGAAGAAGCCGCCCTCGAT
ZM5G852886	*ZmPR1* (S)	TACGGCGAGAACCTCTTCTG
	ZmPR1 (A)	GTTGGTGTCGTGGTCGTAGT
ZM2G441541	*ZmRboh D* (S)	CCGGCTGCAGACGTTCTT
	ZmRboh D (A)	CCTGATCCGTGATCTTCGAAA
ZM5G851266	*ZmPPO1* (S)	AGGCGCTACCGGAAGAGAAC
	ZmPPO1 (A)	GAGAAGTGCACGTCGAACGG
ZM5G811797	*ZmRab7* (S)	TCATCATCCTCGGCGATAGTGG
	ZmRab7 (A)	AGAGCCTGTCCTCGAACTGC
ZM2G326111	*ZmPPI* (S)	ACAAGGGCTCCACCTTCCAC
	ZmPPI (A)	TCGTTGCGCACGAACTTCTC
Zm00001d030028	*ZmMYC7* (S)	CGCCAGTAGCAACAGCACC
	ZmMYC7 (A)	AGCCCTTGAACGGAAACC
Zm00001d043205	*ZmERF147* (S)	CGCCAGTAGCAACAGCACC
	ZmERF147 (A)	AGCCCTTGAACGGAAACC

ZM2G077316	*ZmAOC1* (S)	GTACCTGACCTACGAGGAGT
	ZmAOC1 (A)	GACGATCTGGTTGAGCTTGA

* S: Sense, A: Antisense.
